# From dengue outbreaks to endemicity: Reunion Island, France, 2018 to 2021

**DOI:** 10.2807/1560-7917.ES.2023.28.29.2200769

**Published:** 2023-07-20

**Authors:** Muriel Vincent, Marie Claire Paty, Patrick Gerardin, Elsa Balleydier, Aurélie Etienne, Jamel Daoudi, Fabian Thouillot, Marie-Christine Jaffar-Bandjee, Clinical Investigation Team, Laboratory Network, on behalf of the study collaborators Réseau de médecins sentinelles de la Réunion, Luce Menudier, Clinical Investigation Team, Julien Cousty, Claire François, Loraine Gauzere, Remy Girerd, Françoise Josse, Yatrika Koumar, Marine Lafont, Marie Lagrange-Xelot, Marie-Pierre Moiton, Katia Mougin-Damour, Gil Mourembles, Quentin Richier, Laboratory Network, Nathan Tetart, Mahery Ramiandrisoa, Audrey Pignolet, Cynthia Pianetti, Thibault Bertolotti

**Affiliations:** 1 Santé Publique France, Saint Denis, Réunion, France; 2 Santé Publique France, Saint Maurice, France; 3 INSERM CIC1410, Saint Pierre, Réunion, France; 4 Service de Lutte Anti-Vectorielle, ARS Réunion, Saint Denis, Réunion, France; 5 Laboratoire de Microbiologie et CNR associé des arbovirus, CHU de la Réunion, Saint Denis, Réunion, France; 6 The members of the Clinical Investigation Team are listed under Collaborators; 7 The members of the Laboratory Network are listed under Collaborators; 8 https://sentiworld.sentiweb.fr/network/16

**Keywords:** Dengue, Endemisation, Severity, Reunion Island, Surveillance

## Abstract

**Background:**

After 40 years of limited viral circulation, Reunion Island has since 2018 experienced recurrent dengue outbreaks of increasing intensity and severity.

**Aim:**

We aimed to report on the epidemiology and characteristics of dengue in Reunion Island between 2018 and 2021.

**Methods:**

Between 2018 and August 2021, we systematically collected data on dengue cases via an automated transmission system between the health authorities and the medical laboratories. We set up additional surveillance systems for dengue-related activity in primary care, in emergency departments and in inpatient departments.

**Results:**

Until 2020, despite numerous cases, outbreaks had a limited public health impact because of few severe cases, low lethality and no heavy burden for the health care system. In 2021, however, the number of severe cases increased (from 0.4% of all cases in 2018 to 0.8% in 2021), as did the number of paediatric cases (from 8% in 2018 to 15% in 2021) and atypical clinical forms of dengue (108 cases of post-dengue maculopathy). Of note, haemorrhagic forms were rare and multi-organ failure was the most frequent severity throughout the study period. In parallel, the dominant serotype switched from DENV2 to DENV1 in 2020 and DENV1 became the only serotype detected in 2021.

**Conclusion:**

These findings indicate that dengue is becoming endemic in Reunion Island. Since comorbidities associated with severity of dengue are common in the population, health authorities should carefully consider the impact of dengue when addressing public health policies.

**Key public health message**

**What did you want to address in this study?**

After four seasonal outbreaks, we wanted to address if, in Reunion Island, dengue, a mosquito-borne disease, showed specific characteristics as compared to other French territories. We also wanted to describe the health impact and the transmission dynamics on the island.

**What have we learnt from this study?**

Until 2018, dengue was only sporadically diagnosed in Reunion Island. Between 2018 and 2021, annual outbreaks occurred, leading to a low but constant presence of the virus with the risk of recurrent outbreaks. The outbreaks had an important impact on the health system with increased number of visits to general practitioners and emergency departments. Of note, hypertension and diabetes, common on the island, increase the risk of more severe disease.

**What are the implications of your findings for public health?**

In the Indian Ocean region, dengue should be included in public health strategies on prevention, surveillance, vector control and access to healthcare. Rapid and efficient information sharing between states in the region on circulation of the virus and outbreaks is essential. Travellers should be informed about the risks of import of the virus to Reunion Island as well as the risk of export to Europe in areas where the vector is present.

## Introduction

Over the past 50 years, the incidence of dengue, a viral disease transmitted by *Aedes* mosquitoes, rapidly increased to become the most prevalent arboviral infection worldwide. In the 2010s, the number of notified cases and incidence have been increasing notably in hyperendemic regions such as Oceania, Asia but also in middle and high-middle income regions [1,2].

Dengue fever is most commonly a mild to moderate acute febrile illness, but the infection with dengue virus can vary from an asymptomatic infection to a life-threatening systemic disease. Approximately 500,000 people annually contract severe dengue (dengue with haemorrhagic manifestations or shock syndrome or organ dysfunctions). Of these, 12,500 (2.5%) cases are fatal [1]. There is no specific antiviral treatment, but supportive care of severe hospitalised cases may reduce the case fatality rate to almost zero. The virus can be divided into four serotypes (DENV1, 2, 3 and 4) circulating in humans, while a fifth (DENV5) is so far restricted to sylvatic cycles in Malaysia [3]. After the infection, immunity is life-long against the same serotype but short-lived against others and the risk of developing a severe form increases in secondary infections and with a longer interval between infections [4,5]. Vector control and individual protective measures against mosquito bites are the main preventive options. The European Medicines Agency (EMA) recently authorised a live-attenuated vaccine, QDENGA, developed by Takeda (Tokyo, Japan) based on the DENV2 virus which may not require prior infection in contrast to Dengvaxia (Sanofi Pasteur, Lyon, France), authorised by the EMA since 2018 [6,7].

Reunion Island is a French overseas department in the Indian Ocean. Its population is around 850,000 people and increasing fast. Its subtropical climate allows year-long survival of mosquito populations. *Aedes albopictus* is the main dengue virus vector while *Ae. aegypti* has become very rare on the island. Abundant rainfalls during summer (from December to April) are common in the eastern part while the western part is drier.

After a large DENV2 outbreak in 1977–78 [8], followed by 40 years of low transmission characterised by sporadic cases in summer, dengue virus transmission was first reported again in winter 2017. Since then, the island has seen larger seasonal outbreaks. We describe the epidemiology and characteristics of dengue from 2018 to 2021 in Reunion Island and discuss future challenges and perspectives.

## Methods

The arbovirus surveillance system in Reunion Island is described elsewhere [9]. Briefly, dengue is a notifiable disease and health authorities recommend testing each person with dengue-like symptoms i.e. acute fever associated with one or more of the following signs or symptoms: nausea, vomiting, rash, headache, retro-orbital pain, myalgia, arthralgia or haemorrhagic signs. Positive test results are automatically transmitted from laboratories to the databases of the health authority and classified as described (Box).

Box **Definitions of dengue cases and notification rate, Reunion Island, 2018–2021^a^**

**Confirmed case**

- PCR positive or seroconversion

**Probable case**

- Serological reaction: IgM-positive/IgG-negative in early infections or IgM-positive/IgG-positive when testing is performed > 14 days after symptom onset

**Confirmed secondary infection**

- DENV PCR, positive on two different blood samples, at least 3 months apart or
- DENV PCR positive and specific DENV IgG on the same blood sample

**Probable secondary infection**

- DENV PCR positive on the first sample, followed by DENV IgM and IgG on the second sample, at least 3 months later or
- DENV IgM (with or without IgG) on the first sample, followed by a positive DENV PCR on the second sample, at least 3 months later

• The minimum sampling interval required to consider a secondary infection is 3 months based on Bhoomiboonchoo et al. [29]

**Notification rate**

- The annual number of dengue cases reported in a municipality divided by the population size

^a^ Unless otherwise stated dengue cases refer to both confirmed and probable cases.

This allows an epidemiological follow-up almost in real time and makes it possible to locate emerging clusters of viral circulation precisely and quickly and implement targeted vector-control measures as long as viral circulation is still limited.

The National Reference Laboratory for arboviruses in Reunion Island performs surveillance of dengue serotypes for almost all cases during interepidemic phases and on a geographically representative selection of samples during epidemic phases. When possible, all imported cases, severe and/or atypical cases and fatal cases are serotyped.

An additional surveillance system estimates the total number of cases with dengue-like syndrome during epidemic periods, based on the sentinel network of general practitioners. This network reports weekly the number of cases with dengue-like syndrome to Santé publique France. Coupled with the total weekly number of medical consultations transmitted by the National Health Insurance, the total number of cases with dengue-like syndrome is extrapolated.

The surveillance of dengue-related activity in emergency departments (ED) for adults and children relies on the automated ED surveillance by the Organisation de la Surveillance Coordonnée des Urgences (OSCOUR) network. The surveillance of dengue hospitalisations (> 24 h) is conducted in the four regional hospitals (Centre Hospitalier Universitaire site north and south, Groupement Hospitalier Est Réunion and Centre Hospitalier Ouest Réunion). It aims to monitor epidemic severity by collecting clinical data such as the presence of warning and severity signs [9,10]. Risk factors for severe dengue i.e. pre-existing renal and hepatic failures, diabetes, pregnancy, age below one year and above 75 years, platelet disorders, sickle cell anaemia and social isolation, are also collected.

Surveillance of post-dengue maculopathy started in 2020 after the emergence of first cases. Clinicians report on post-dengue maculopathy cases seen in hospitals or in primary health care practice and laboratory results and clinical information are centralised in a common database.

A dedicated committee analyses all fatal dengue cases and assesses the causal role of dengue based on medical data and the algorithm developed in French Antilles [9].

Data presented in this report cover the period between 1 January 2018 and 31 August 2021 (week 35). Besides median age comparisons, age groups were chosen as follows: 0–14 years, 15–29 years, 30–44 years, 45–59 years, 60–75 years and > 75 years.

For statistical analysis, Pearson’s chi-squared test was performed to compare frequencies of occurrence of several observations. The Pearson correlation coefficient was calculated to assess correlation between the number of cases with dengue, cases with dengue-like syndrome and of ED visits for dengue-like syndrome (https://www.socscistatistics.com/tests/chisquare2/default2.aspx).

## Results

### Outbreaks

**Figure 1:**
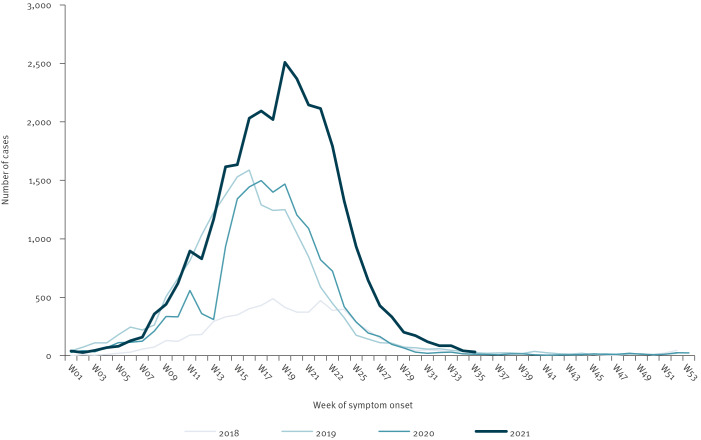
Number of confirmed dengue cases per week, by onset of symptoms, Reunion Island, France, 2018–week 35 in 2021 (n = 70,724)

**Figure 2:**
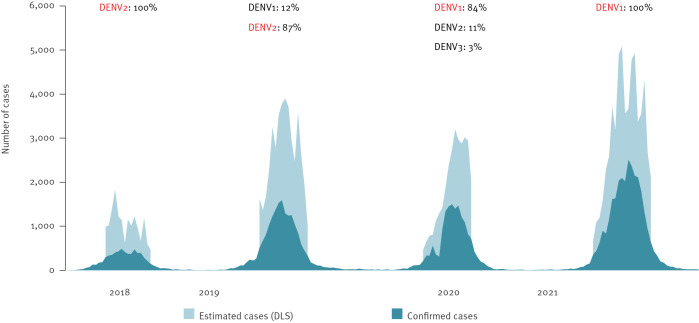
Number of estimated dengue cases and confirmed cases, Reunion Island, France, 2018–week 35 in 2021 (n = 147,686)

Between 2018 and 2021, four seasonal dengue outbreaks of increasing intensity were detected in Reunion Island. These outbreaks were characterised by a fast rise in cases end-February/beginning of March (week 8 and 9), a peak between mid-April and mid-May (between week 16 and 19), followed by a rapid decrease in July (week 24 and 25) coinciding with the onset of the southern hemisphere winter (from June) (Figure 1). The number of cases notified and the estimation of cases with dengue-like syndrome strongly correlated through the study period, indicating that COVID-19 did not significantly interfere with this surveillance (r = 0.8 in 2020 and 0.9 in 2021 vs 0.8 in 2019) (Figure 2).

The notification rate of dengue cases rose from 784 per 100,000 in 2018 to 3,417 per 100,000 inhabitants in 2021 (Table 1).

**Table 1:** Characteristics of dengue outbreaks, Reunion Island, France, 2018–week 35 in 2021 (n = 4)

Characteristics	2018	2019	2020	2021^a^
Notified incidence (number of cases/100,000 inhabitants/year)	784	2,110	1,870	3,417
Epidemic season (weeks)	W13–27	W9–24	W8–23	W8–26
Week with most notifications	W18	W16	W17	W19
Number of cases in peak week	488	1,588	1,498	2,509
Autochthonous cases	6,770	18,217	16,141	29,596
Number of dengue-like syndromes (epidemic period only)	15,464	42,415	30,575	59,232

W: week.

^a^ Up to week 35.

In 2020, the number of cases decreased at the onset of the COVID-19 pandemic and the lockdown of March and April (Figure 1).

The outbreak in 2021 was the largest with more than 2,500 weekly notified cases at peak and almost 30,000 cases in total. Approximately 5,000 people with dengue-like syndrome were notified at peak and almost 60,000 in total. For the first time, dengue was reported in more than 10% of the population in two towns in the western part of the island. In parallel, the viral circulation increased in the north: the notification rate increased from three- to fivefold in two municipalities compared with previous years.

The outbreak lasted longer (18 weeks), and the weekly number of cases remained higher during winter than in previous years. Altogether, during the surveillance of 4 years, 150,000 cases with dengue-like syndrome were notified, almost twice the number of cases. Also, the number of PCR tests performed and the proportions of test-positive results increased (n = 23,864; 28% in 2018 to n = 68,013; 38% in 2021).

**Figure 3:**
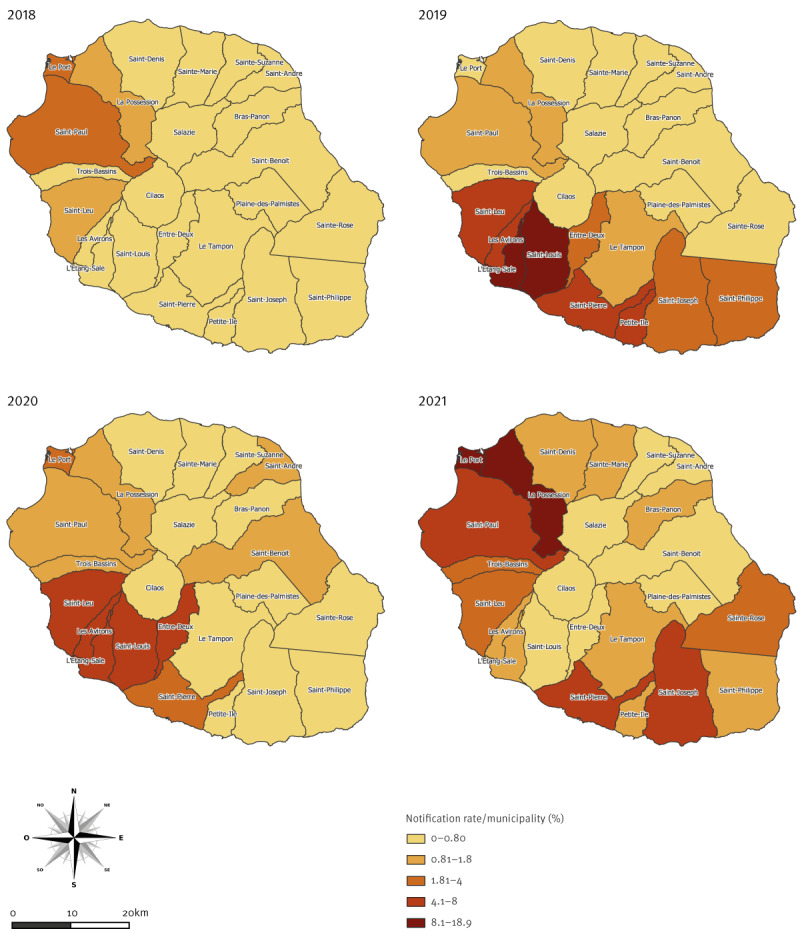
Dengue notification rate, per year and municipality, Reunion Island, France, 2018–week 35 in 2021

The west of the island was the most affected area in 2018 and 2021, the south in 2019 and 2020, while circulation remained lower in the north and the east in the period between 2018 and 2021 (Figure 3).

### Dengue serotypes

Most dengue cases were diagnosed using PCR: 72% (n = 13,177) in 2019 and 83% (n = 13,406) in 2020, while 28% (n = 5,040) in 2019 and 18% (n = 5,329) in 2021 were diagnosed with serology.

The proportion of serotyped PCR-positive samples decreased from 18% (n = 950/5,356) in 2018 to around 5% (n = 978/24,267) in 2021. During the study period, more than 90% (n = 3,358/3,649) of the serotyped samples were analysed for surveillance purposes, while severe, atypical and fatal cases were a minority and fewer than 1% (n = 28/3,649) were from imported cases.

In 2018, DENV2 was the only serotype among autochthonous cases. DENV1 was first isolated in 2019 in an autochthonous case after an introduction in the southern part of the island from an imported case. When the outbreak restarted in 2020, DENV1 expanded to the west and became dominant by the end of the year. DENV3, first identified in an autochthonous case at the end of 2019, represented 3.5% (n = 29) of all serotypes in 2020 but remained limited to the eastern region and was no longer detected in 2021.

Co-infections with DENV1 and DENV2 were detected in four patients in 2019 and in three in 2020.

### Sex and age distribution

The male: female rate was stable among the infected. The median age among cases ranged from 38–42 years (Table 2). Over time, the proportion of cases in younger age groups (< 15 years and 15–29 years) increased significantly in contrast with 30–59-year-old cases. The proportion of cases in the age groups over 60 years remained stable, ranging from 18 to 22% (Table 2).

**Table 2:** Demographic characteristics of dengue cases, Reunion Island, France, 2018–week 35 in 2021 (n = 70,617)

Characteristics of dengue cases	2018			2019		2020		2021^a^		p value (2018 vs 2021)
	n		%	n	%	n	%	n	%	
Dengue cases										
Sex ratio (male/female)	1.1			0.9		1.1		1.1		NA
Median age in years (range)	42 (0–96)			42 (0–97)		40 (0–98)		38 (0–100)		
Total	6,758^b^			18,142^b^		16,121^b^		29,596^b^		
0–14 years	555		8	1,724	10	1,870	12	4.298	15	≤ 0.0001
15–29 years	1,214		18	3,439	19	3,144	20	6,412	22	≤ 0.0001
30–44 years	1,880		28	4,716	26	4,186	26	7,160	24	≤ 0.0001
45–59 years	1,788		27	4,351	24	3,849	24	6,390	22	≤ 0.0001
60–75 years	1,056		16	2,903	16	2,505	16	4,390	15	NS
> 75 years	265		4	1,009	6	567	4	946	3	≤ 0.01
Secondary infections	0			22	0.1	1,112	7	5,139	17	NA
Dengue cases hospitalised for more than 24 h										
Total	154	2		620	3	787	5	1,111	4	≤ 0.0001
0–14 years	7	0.1		23	0.1	36	0.2	75	0.3	NS
15–29 years	29	0.4		78	0.4	76	0.5	132	0.4	NS
30–44 years	15	0.2		82	0.5	110	0.7	154	0.5	≤ 0.0001
45–59 years	36	0.5		90	0.5	182	1.1	216	0.7	≤ 0.01
60–75 years	42	0.6		177	1.0	244	1.5	363	1.2	≤ 0.0001
> 75 years	25	0.4		170	0.9	122	0.8	171	0.6	p ≤ 0.001
Dengue cases hospitalised for a severe form										
Total	27	0.4		75	0.4	108	0.7	245	0.8	p ≤ 0.001
0–14 years	1	0.01		3	0.02	2	0.01	13	0.04	NS
15–29 years	0	0		5	0.03	8	0.05	28	0.09	≤ 0.05
30–44 years	1	0.01		4	0.02	18	0.1	41	0.1	≤ 0.01
45–59 years	10	0.6		14	0.08	27	0.2	49	0.2	NS
60–75 years	9	0.9		23	0.1	38	0.2	82	0.3	≤ 0.05
> 75 years	6	2.3		26	0.1	15	0.09	32	0.1	NS
Case fatality										
Number of dengue-related deaths	6			14		22		33		NA

NA: not applicable; NS: not significant.

^a^ Up to week 35.

^b^ Age was not available for all cases; missing values for 2018: 12, 2019: 75, 2020: 20.

### Secondary dengue

The first secondary infections were recognised in 2019 with the emergence of DENV1. In 2021, 5,139 (17%) cases had secondary infections. Most of them (n = 4,981; 97%) were confirmed secondary cases and 81% of them (n = 4,173) had a DENV PCR- and IgG-positive result. The median interval between the primary and secondary infections was 13 months in 2019 and 2020 and 33 months in 2021. The shortest interval reported was 63 days between two positive PCR tests, while the longest was 52 months. In 2020 and 2021, among the secondary cases, only 19% were younger than 30 years (1,190/6,251), while 37% (14,534/39,466) of the primary cases belonged to this age group (p < 0.00001).

### Dengue severity and burden on healthcare

#### Dengue-related cases in emergency departments

The dengue-related activity in ED correlated strongly (correlation coefficient 0.7–0.9 during the study period) with the number of cases and the outbreak intensity, but also temporally and spatially.

While the overall ED activity remained stable, the number of dengue-related visits to the ED increased from less than 500 visits in 2018 to more than 4,000 in 2021 (a 10-fold increase from 0.3% to 3.4% in ED activity).

#### Annual dengue related hospitalisations and severe cases

The number of hospitalised cases increased from 154 in 2018 to 1,111 in 2021, a rise from 2% to 4% of all cases. As for the total number of dengue cases, there was no difference in hospitalisation rates between males and females. However, the median age of hospitalised cases was higher, around 60 years. During the study period, the proportion of hospitalised cases younger than 15 years increased from 0.1% to 0.3% but older cases (above 60 years) were more frequently hospitalised (Table 2).

The proportion of severe cases increased significantly, from 0.4% in 2018 and 2019 to the double (0.8%) in 2021 (Table 2). This increase was seen in all age groups. The proportion of severe cases admitted to an intensive care unit decreased from 40% (n = 62) in 2018 to 22% (n = 244) in 2021.

Detailed clinical information was available for 354 of the 455 severe cases. A total of 274 severe dengue cases had comorbidities: hypertension (n = 213; 78%) was most common, followed by diabetes (n = 140; 51%). A total of 291 severe dengue cases had warning signs: thrombocytopaenia was the most common (60%; n = 174). Organ failures were more frequent (n = 242; 68%) than severe plasma leakage (n = 82; 23%) and severe bleeding (n = 15; 4.2%). Kidney and liver failure were the most common complications among severe cases. Of note, 22 subjects presented more than one severity sign.

We serotyped 180 (59%) of the 307 isolates from severe dengue cases confirmed with PCR. The clinical picture did not differ between cases with various serotypes, however, the sample size was too small for statistical analysis (data not shown).

The proportion of severe cases was significantly higher in patients with secondary episodes than for those with first episodes: 30% (n = 60) vs 20% (n = 326); p value = 0.0015).

#### Ophthalmic complications

In 2020 and 2021, for the first time in Reunion Island, 126 patients were reported with post-dengue maculopathy. These patients represented 0.1% (n = 18) and 0.4% (n = 108 patients) of the notified cases in 2020 and 2021, respectively. Maculopathy was more often diagnosed in females (n = 84; 67%) than in males (n = 42; 33%). In 2021, 77% (n = 83) of the maculopathy cases were between 15 and 44 years. The ophthalmic symptoms started a median of 7 days after the onset of dengue symptoms. The only serotype identified in these cases was DENV1. Patients with post-dengue maculopathy had more often a secondary infection (n = 24: status of the infection was known for 71 patients) compared with the total number of cases in 2021 (p = 0.006).

#### Case fatality associated with dengue

In the period 2018 to 2021, 75 (0.1%) of the 70,724 notified dengue cases died. Infection status (primary infection vs secondary infection) was available for 51 fatal cases. Case fatality was significantly higher in secondary forms (0.22% vs 0.09%; p = 0.004). Fatal cases were older than the cases in general: median ages were 72 and 40 years, respectively. Most fatal cases had a severe form of dengue (information was only available for hospitalised cases). The death of 43 cases was directly linked to dengue.

Old age (> 75 years) and pronounced lethargy were significantly more common in severe dengue cases with fatal outcome than in non-fatal severe cases (Table 3).

**Table 3:** Characteristics of fatal and severe dengue cases, Reunion Island, France, 2018–week of 35 in 2021 (n =400)

Characteristics of dengue cases	Severe cases (n = 354)^a^		Fatal cases (n = 46)^a^		p value
	n	%	n	%	
Cases with risk factors	274		34		
Cases > 75 years	69	25	17	50	≤ 0.01
Cases with warning signs	291		35		
Cases with pronounced lethargy	130	45	23	66	≤ 0.05

^a^ Detailed clinical information was not available for all severe and fatal cases.

Other clinical features were comparable between severe and fatal cases. Death generally resulted from multi-organ failure in people with multiple comorbidities. In 2020, 18 of the 22 fatal cases had multiple comorbidities when 19 of the 33 fatal cases in 2021 had them. In addition, four women younger than 50 years died unexpectedly with cardiac failure without any known risk factors for severe dengue.

## Discussion

The United States Centers for Disease Control (CDC) defines an endemic disease as “the constant presence of a disease or infectious agent within a geographic area or population group” [11]. For dengue, there are additional characteristics of endemicity to consider such as increased number and proportion of paediatric cases, the severity of the illness, the emergence of atypical clinical presentations of the disease, switch between serotypes and periodicity of outbreaks every 2–5 years [12-15]. Surveillance data from Reunion Island are consistent with these characteristics and, coupled with a sustained low-level circulation and the absence of outbreak in 2022, they support the fact that dengue is now endemic in Reunion Island.

Outbreaks affected the four regions differently, with the largest impact on the south-western region and limited effect on the north-east. The southern and the western areas of the island are drier, hotter and more densely populated, while the east has a lower population density possibly limiting the transmission. Also, heavy rainfalls in the eastern part contribute to lower mosquito densities [16].

Most of the severe cases had organ impairment. Classically, plasma leakage and haemorrhagic manifestations are more frequent than severe organ impairment, mainly described in people with underlying conditions [17,18]. The high prevalence of hypertension, diabetes, renal failure and of cardiovascular diseases in Reunion Island, all risk factors of severe dengue [19], may be an explanation. In parallel, the case fatality remained low (0.1% of all cases), as observed in countries with similar healthcare systems [1,4], and fatal cases occurred mostly in secondary forms and in people with multiple comorbidities.

In 2021, the total disappearance of serotypes DENV2 and DENV3 was unexpected; such sudden development is rarely reported in the literature (except for DENV4) [12]. Herd immunity against DENV2 is probably not reached everywhere and in addition, population movements bring every year naïve people, prone to the infection, to the territory. Regarding DENV3, its limited presence in 2020 (less than 5% of the serotyped samples and only from the eastern region) does not fully explain its disappearance in 2021. However, the proportion of serotyped samples decreased over time and became very low (5% in 2021), which could explain why DENV2 and DENV3 were not detected. A limitation of our work lies in the few isolates serotyped and missing genotyping. However, in the context of COVID-19 pandemic, sequencing techniques adapted to dengue have become available locally. Genotype data should therefore be more widely available soon.

Data on post-dengue maculopathies are limited but the literature suggests that they could emerge after a switch of a dominant serotype [20]. In Reunion Island, these forms were associated with DENV1, and secondary infections and eye symptoms mainly occurred 7 days after the onset of dengue symptoms, at the nadir platelet count. These associations have been reported by others as well, however, in contrast to previous reports, in Reunion Island, young females were predominantly affected [20-22].

Since 2018, the surveillance of dengue has been regularly adapted following epidemiological and clinical evolutions. This adaptability is a strength for the collection of data of good quality. The collaborative work with clinicians, coupled with automation in the transmission of data, improved the robustness and exhaustiveness of hospital data. We established practical definitions for probable secondary infections and, while they differ from classifications based for example on IgM/IgG ratio [23], we applied the same definition to all data for robustness.

Dengue prevention relies on the reduction of contacts between humans and the vector. A vector control strategy includes sanitation measures, improvement of housings conditions, reduction of waste and open water containers and antivectorial treatments. The inaccessibility of some breeding sites and the presence of open water storage containers in gardens, however, hamper the efficacy of anti-vectorial treatments. For instance, mosquito densities are higher in the drier western and southern regions where the use of water containers is common [16]. On the other hand, the use of insecticide is associated with vector resistance, toxicity or social reluctance, leading to a strong need for innovative vector control techniques. In the near future, supported by positive results of field experiments [24-26], the deployment of the sterile insect technique could benefit the territory by helping to reduce vector density. At individual level, the use of repellents and mosquito nets should be strengthened through social mobilisation. Finally, vaccine recommendations for prevention may evolve after the authorisation of the live-attenuated QDENGA vaccine which may not require a proof of prior infections like the Dengvaxia vaccine does.

Prevention should also address the burden of some chronic diseases and risk factors for severe dengue, such as diabetes, hypertension and chronic renal failure, which contributed to the high proportion of organ impairment observed in severe cases in our study, in contrast to other territories affected by dengue [4,17]. Regarding the access to healthcare, the outbreak of 2021 had a large impact on emergency departments and inpatient activity. Health authorities must consider that a similar outbreak might emerge again when allocating resources to hospitals.

In the south-western Indian Ocean region, despite limited data, there is evidence that from the Seychelles to Madagascar or to the Comoros, dengue outbreaks occur regularly, and the disease is becoming endemic [27]. New dengue viruses may emerge in the Reunion Island. International collaboration on epidemiological surveillance through the SEGA-One Health network (https://umr-astre.cirad.fr/en/research/projects/sega-one-health) should be strengthened to enhance dengue surveillance coordination. A seroprevalence study in Reunion Island would give information on the performance of the epidemiological surveillance by comparing seroprevalence with surveillance data.

Lastly, dengue endemisation in Reunion Island, another French overseas department affected by dengue together with the French Antilles and Guyana, is also a challenge for mainland France. Connections between overseas departments and mainland France are numerous and 64% of all imported dengue cases in France in 2021 were from the island. Hence, dengue epidemics in Reunion Island are a concern also for mainland France, where the vector *Ae. albopictus* is well established and local transmission now occurs every year [28].

## Conclusions

With the endemisation of dengue and the plausible occurrence of future outbreaks, dengue prevention and control need to be integrated in the Reunion Island public health strategy. This should particularly be the case when addressing comorbidities, such as diabetes or hypertension, which are risk factors for severe dengue and very common on the island. In addition, the surveillance of serotypes and genotypes should be reinforced to quickly detect the introduction of new viral strains. In parallel, new vector control strategies positively tested locally could benefit the whole island.

Finally, increased awareness is needed for travellers to mainland France: local transmission could start after introduction of imported cases in areas where *Ae. albopictus* is established. Clinicians should consider dengue diagnosis when investigating fever, especially in travellers. The recent outbreaks in Reunion Island highlights the continuing expansion of dengue worldwide. In this context, an integrated strategy encompassing increased awareness, social mobilisation, coordinated surveillance and the development of innovative vector control techniques is of paramount importance.
